# Personal health care of internal medicine residents

**DOI:** 10.3402/jchimp.v1i4.8864

**Published:** 2012-01-26

**Authors:** Venkataraman Palabindala, Paul Foster, Swetha Kanduri, Avanthi Doppalapudi, Amaleswari Pamarthy, Karthik Kovvuru

**Affiliations:** Greater Baltimore Medical Center, Towson, MD, USA

**Keywords:** mailed questionnaire, confidentiality, primary care, health access

## Abstract

**Introduction:**

Medical residents, as part of their job to balance the demands of their work with caring for themselves so as to be mentally, emotionally, and physically sound to stay clinically competent. While regulatory and legislative attempts at limiting medical resident work hours have materialized but have yet to attain passage, there are fairly little data looking into how residents cope up with their demands and yet attend to their own personal health.

**Design:**

Anonymous mailed survey.

**Subjects:**

Three hundred and thirty-seven residents from all internal medicine residency programs within United States.

**Methods:**

We conducted a survey in the form of a questionnaire that was sent by e-mail to the program directors of various internal medicine residency programs within the United States, and responses were collected between May 19 and June 21, 2009. Response was well appreciated with total number of participants of 337 with even demographical distribution in gender, residency year, AMG/IMG, age group. Seventy-one percent of the residents felt that they would prefer getting admitted to their own hospital for any acute medical or surgical condition. Of the 216 residents who have had received health care in the past, almost half of them chose their own hospital because of the proximity, while 45% did not choose their own hospital despite proximity. Two out of three residents missed their doctors appointments or cancelled them due to demands of medical training. Only half of the residents have a primary care physician and almost 80% of them did not have their yearly health checkup. Close to 30% held back information regarding their social and sexual history from their provider because of privacy and confidentiality concerns. Eighty percent of residents never received information about barriers that physicians may face in obtaining care for their socially embarrassing conditions. Seventy percent felt that their performance then was suboptimal because of that health condition and also felt sick but did not drop the call. Half of the residents had concerns that they might be having a psychiatric illness, but only 5% of them received a formal evaluation at their own hospital and 23 (12.4%) at an outside hospital.

**Conclusions:**

It is very important to have more studies to emphasize on resident's physical and mental health and encourage them to have a primary care physician. There are several reasons preventing residents from getting a formal evaluation, confidentiality reasons, lack of time – schedule constraints, fear of being labeled, and social repercussions are few of them. Program directors should encourage the residents to not only care of the health of their patients but also be enthusiastic about their personal health issues for upgraded, revised patient care, and ultimately for their overall well-being.

Medicine as a profession needs to be civilized with dedication, patient care, humanity, and cultural integrity (1). Physician generally behaves like the legends in providing services to the patients. Focusing on residents during their training period, approximately every year around 100,000 physicians participate in the residency training in all the institutions across the United States. In the process of providing finest care, they may be suffocated with various overlapping roles as a trainee, provider, and a counselor. This multitasking may leave the residents susceptible to various physical, mental, and emotional health strategies.

The death of an 18-year-old college student Libby Zion, in the New York city who was under the care and attention of a terribly fatigued resident, was incredibly worrisome (2) and this aroused the significance of residents health factors, which is now proven to be playing a pivotal role in their overall personality development. Verghese has commented on the physicians’ isolated and deserted lives that ‘the measure of the health of our profession is not only how well we care for our patients but also how well we care for ourselves.’(3).

Medical residencies traditionally require lengthy hours of their trainees. The medical education establishment is increasingly recognizing that such long hours are counter-productive, because sleep deprivation increases rates of medical errors. This was noted in a landmark study on the effects of sleep deprivation and error rate in an intensive care unit. Most recently, the Institute of Medicine (IOM) built on the recommendations of the ACGME on resident duty hours: enhancing sleep, supervision, and safety. Although these limits are voluntary, adherence has been mandated for the purposes of accreditation.

As the residents have to go through this laborious lengthy days during the training process and deal with the extraordinary challenges, their own physical and mental health needs may increase tremendously (4). The report also suggests residents be given variable off-duty periods between shifts, based on the timing and duration of the shift, to allow residents to catch on sleep each day and make up for chronic sleep deprivation on days off. Research from Europe and the United States on non-standard work hours and sleep deprivation found that late-hour workers are subject to higher risks of gastrointestinal disorders, cardiovascular disease, breast cancer, miscarriage, preterm birth, and low birth weight of their newborns.

## Study methods

We conducted a survey in the form of a questionnaire that was sent by e-mail to the program directors of various internal medicine residency programs within the United States with instructions to forward this to their residents. Responses were collected between May 19 and June 21, 2009.

## Results

Although a total of 349 started the survey, only 337 (96.6%) completed it. Of them, 179 (51.6%) were male and 168 (48.4%) were female. The majority were between the ages of 20–30 at 225 (64.7%); 112 (32.2%) were between the ages of 30–40. 9 (2.6%) were between 40–50 years of age; 2 (0.6%) were above 50 years of age. One hundred and thirty-four (38.7%) were PGY1; 116 (33.5%) PGY2; 92 (26.6%) PGY3; 4 (1.2%) PGY4. One hundred and seventy-three (49.7%) were International medical graduates, whereas 175 (50.3%) were American Medical Graduates. One hundred and sixty-seven (48.1%) worked the most in a university hospital and 180 (51.9%) worked the most in a community hospital setting. These demographics are shown in Table 1.

**Table 1:** Demographics of the study

Gender	Male	179(51.6%)	Female	168(48.4%)
Age	20–30	30–40	40–50	>50
	(225) 64.7%	(112) 32.2%	(9) 2.6%	(2) 0.6%
Year of residency	PGY1	PGY 2	PGY3	PGY 4
	(134) 38.7%	(116) 33.5%	(92) 26.6%	(4) 1.2%
Are you IMG or AMG	International	(173) 49.7%	American	(175) 50.3%
Type of hospital do you work	University hospital	(167) 48.1%	Community hospital	(180) 51.9%

Table 2 describes further characteristics of the respondents that if they had an acute abdomen, which hospital would they prefer for possible admission? Out of 337 residents, 245 (71.2%) felt they would prefer getting admitted to their own hospital. However, 99 (28.8%) preferred any hospital other than their own for an acute hospital admission. Of the 216 residents who have had received health care in the past, 120 (55%) chose their own hospital because of the proximity, while 96 (44.4%) did not choose their own hospital despite proximity; 230 (67.1%) missed their doctors appointments or had to cancel them because of their busy schedule or demands of medical training; 113 (32.9) made it to their doctors’ appointments without difficulty. Out of 344, only 183 (53.2) have a primary care physician and a staggering 161 (46.8%) have not established care yet with a primary care physician. Out of 342, a majority of 270 (78.9%) did not have their yearly health checkup, while 72 (21.1%) saw their PCP at least on a yearly basis.

**Table 2:** Do you have a doctor?

	Yes	No
Have primary care	183 (53.2%)	161 (46.8%)
Missed appointments due to busy schedule in residency	230 (68.8%)	113 (31.2%)
Attended yearly physical	72 (21.1%)	270 78.9%
Preventive medicine and immunization effected by busy schedule	158 (47.6%)	174 52.4%

Out of 332 although a majority of 238 (71.7%) did not have a problem, 94 (28.3%) held back information regarding their social and sexual history from their provider because of privacy and confidentiality concerns. While 289 (90.6%) felt that it did not have any impact, 30 (9.4%) of them felt that holding back such information had an impact on the quality of care they received. Table 3 basically describes about such concerns.

**Table 3:** My Privacy? I'm concerned!!

	Yes	No
Did you ever hold back information related to your social or sexual history from your provider because of privacy or confidentiality concerns?	94 (28.3%)	238 (71.7%)
Did revealing (or not revealing) your accurate social and sexual history impact the quality of care that you received?	30 (9.4%)	289 (90.6%)

Fifty-five (16.6%) out of 330 experienced personal health care-related incidents with a breach in confidentiality. For a question whether they received information about barriers that physicians may face in obtaining care for their socially embarrassing conditions, 261 (79.3%) never received any such. However, 45 (13.7%) report to getting such information from their residency program, 6 (1.8%) from employee health, and 17 (5.2%) from Internet/web. This information has been illustrated in Table 4.

**Table 4:** How many times are you worried?

Have you experienced any personal health care-related incidents where you suspected a breach in confidentiality?	
Once or twice	14.5% (48)
Three to four times	0.6% (2)
Greater than five times	0.0% (0)
Always	1.5% (5)
Never	83.3% (275)

**Fig. 1:**
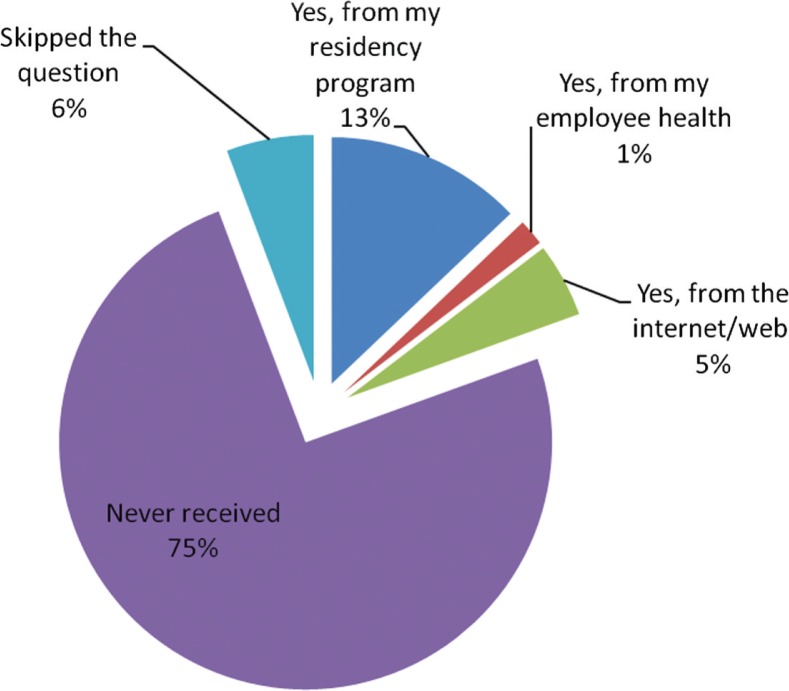
Information about barriers that physicians may face in obtaining care for their socially embrassing conditions.

Figure 1 shows that since starting residency, 173 (51.8) fell sick enough to drop their call but never actually did for at least once or twice. Eighty-four (24.1%) fell sick enough to drop their call for three or more times but never actually did. Only 77 (23.1%) did not fall sick enough to drop their calls during residency. One hundred and eighty-four (71%) felt that their performance then was suboptimal because of that health condition.

**Fig. 2:**
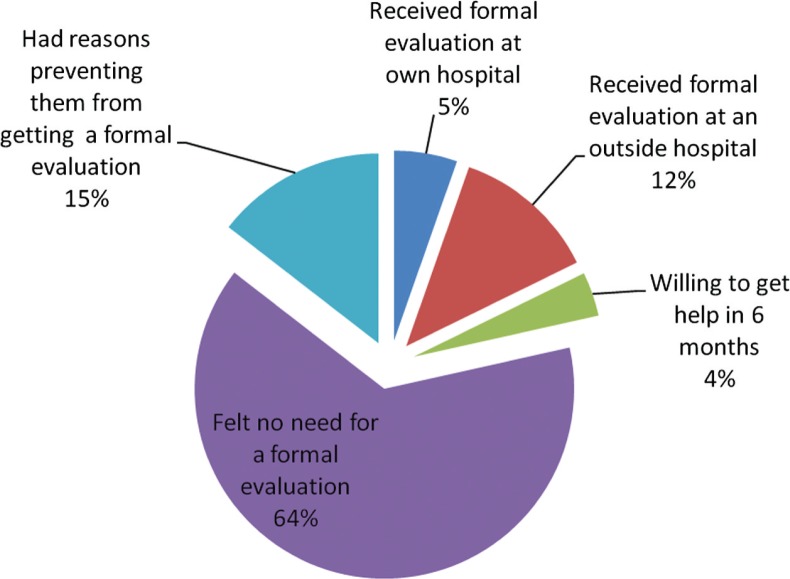
55% of 334 had concerns of Depression/Psychiatric illness.

Figure 2 shows that Out of 334, 185 (55.4%) had concerns that they might be having depression, anxiety, or a psychiatric illness. Ten (5.4%) of them received a formal evaluation at their own hospital; 23 (12.4%) received a formal evaluation at an outside hospital. Seven (3.8%) are willing to get help in the coming 6 months; 119 (64%) did not feel the need to be formally evaluated; 27 (14.7%) had other reasons preventing them from getting a formal evaluation. Among them were confidentiality reasons, lack of time – schedule constraints, fear of being labeled, social repercussions, and job ramifications.

## Discussion

Residency is one of the most exhausting and the challenging periods in a physician's life. It is quite distinct period during which the physicians may face many hurdles such as sleep deprivation, loneliness, tend to deal with the financial constraints while being traumatized with mental health issues. They need to prioritize their demands and deal with the ambiguity while maintaining the balance in their lives. They may be bugged with atypical and unpredictable schedules and due to this intensive work load their health needs pop up (5–7). This multiplication of various factors creates a sort of health deficit and the significant stress, burden that the residents experience may contribute to their physical and emotional health problems. In the literature, there are only few studies that emphasized on the personal health care of internal medicine residents, the confidentiality issues, and the barriers they overcome in accessing the health care.

Out of 339 residents participated in our study, it is evident that most of the medical residents are young and healthy, and their distribution regarding gender, International Medical Graduates versus American Graduates and residents from community and university-based hospitals, is pretty much even. Fatigue and distress expressed during residency period are shown to be universal and the loneliness, isolation during this period may affect their future health patterns. It is very pathetic that residents getting the regular annual visits to the hospitals lag behind, and this pattern could lead to the behaviors that may ultimately jeopardize the health of our future physicians. Therefore, the residents should have access to the comprehensive medical care to not only to be fit and competent during the training but also to cope up with their future career goals. The programs should come up with explicitly important steps to improve the residents’ attitude regarding appropriate medical and psychological care with due emphasis on the acute and chronic health issues (2).

The fact that most of the residents visit the hospital for their health issues only during acute ill conditions is annoying and the unbelievable response of the residents of visiting their own hospital due to its proximity but not due to their personal emotional affiliation is even more concerning. It has become enormously significant issue to encourage them to attend the clinics, mentor them for appropriate self-care, and emphasize them on autonomy, objective medical care via conferences, support groups, and counseling (8). Empirical studies have shown that the residents being a part of health care system are quite aware of the harmful effects of self-treatment and that the acquisition of health care by them personally is not that extravagant when compared to general population.

Residency is an idealistic period during which their health needs modify vigorously and health status declines. Even then numerous times residents avoid or postpone obtaining health care; they usually miss appointments because of their hectic and unpredictable schedules. On viewing our study results, two-thirds of residents did not show up at the doctor's office, which may show an insight into their intensive work hours and excessive burdens. Only one-third could make up their appointment at the scheduled times. Their concern for privacy, confidentiality, stigmatization, and the attitude of their peers may be the contributing factors (2). The residents often have solicited report that programs and house staff are not sympathetic to their appropriate health care (4). Only around half of the residents have the primary care and the other half does not understand the need for personal family physician. They often informally consult their colleagues, who share the resident's vulnerability and dissatisfaction. Ironically, studies from the United States substantiate that more than 50% of the residents fill their own prescriptions and are obliged to have self-diagnosis and treatment (9). This has been emerging as one of the important barriers preventing them from accessing health care.

The Canadian Medical Association (CMA), the College of Physicians and Surgeons of Ontario (CPSO) elucidated that for all the conditions other than medical emergencies, for themselves or their family members, it is unethical and indecent for the physicians to go with the self-treatment (10). The Professional Association of Interns and Residents of Ontario (PAIRO) emphasize on the fact that it is very appropriate and eligible for each and every resident and the physician to have a personal primary care physician (11). This is one of the most challenging issues that needs to be addressed by the programs actively to avoid the self-negligence, while affirming for the prospective growth and physical well-being of physicians lives.

It is interesting to know that around 80% of the residents do not have yearly health checkups, while 20% of them actually had. National Population Health Survey done in 1994 has proven that the primary health care options such as the preventive strategies are very well received by the physicians who had annual health checkups according to their family physician's recommendations (12). Not surprisingly, the concept of healthier life styles practiced by the physicians has an extraordinary impact on the patient's lives. For example, the physicians who do not smoke or use illicit drug and do exercise regularly have precise impingement in modifying their patient's habits, favoring them to quit smoking, etc.

Most of the residents held information regarding social and sexual history from their providers because of privacy and confidentiality concerns, and they felt that it had no effect on the quality of care they received. From our study, 20% of the residents experienced personal health care-related incidents with a breech in confidentiality. The residents do face some barriers in obtaining care for their socially embarrassing conditions. Measures in overcoming such barriers are usually provided by the residency programs, support groups such as employee health assistance programs (13), and Internet that may help them in battling with these challenging situations. Unbelievably, almost 70% of them never received such information. Therefore, further inquiry is absolutely important to understand how extensively these factors influence the integrated lives of our residents. Programs should strive to inform residents thoroughly about the more stigmatizing conditions, clear up their ambiguities, and educate them regarding their policies and rights related to personal health care at the time of orientation.

The anticipation of potential professional jeopardy, stigma, and the criticism of their colleagues may affect residents ‘health seeking attitudes.’ In our study from the period of beginning of residency, almost 50% of residents felt sick enough to drop their call but never dropped it. Most of them felt that their sickness affected their performance, and it was suboptimal compared to their regular days. They were in heightened fatigue and ongoing emotional distress that jeopardized their health creating a chaotic situation in their lives.

Around half of the medical residents in our study responded that they are bothered about depression or a psychiatric illness during residency training. Literature shows multiple studies conducted in the past revealed that a significant number of the medical residents reported a period of depression during their residency. Residents and physicians may seek attention for acute illness, but the probability of them seeking appropriate care for mental and psychiatric issues is very less even though they are in emotionally vulnerable situations. Unfortunately, the extreme stress may lead to psychological upset with increased number of medical residents and physicians suffering from anxiety, schizophrenia, and bipolar disorders (14). This has indulged in the information that physicians who are emotionally traumatized, their work performance, basic clinical skills have been compromised, and they tend to be inefficient in providing the utmost patient care.

Finally, we would like to present that resident well-being plays a key role in maintaining professionalism. Resident physician plays a very vital role in the lives of people and in the process of saving their lives, they usually neglect their health concerns. It is very transparent that they could provide excellent services and be justified to their job only if they are physically, emotionally sound, and competent. Now it is time for all the residents to have a primary care physician with annual checkups and to go for appropriate screening tests. Programs should come up with seminars, guest lectures, and specific organizations to make their residents overall developed and well-trained professionals. Privacy and confidentially issues should no more be a barrier in accessing the health care.

## Limitations

Like any other studies, our study too has its own limitations. The questionnaire was sent only to the internal medicine residency programs and it did not include any of the other residents of surgery, orthopedics, pediatrics, family medicine, etc. Although this survey includes geographically widespread residents, the fact that only internal medicine residents are involved emphasizes further elaboration of this study with subset and sex-based analysis. Because this is a retrospective study, there is a significant chance for the residents to come up with a recall bias. Even though the study was initially designed to focus on primary care for the residents and confidentiality issues, so many factors are addressed and finally we do lack more specific conclusions from our data. Challenging on many a number of concerns may dilute the initial obligations of the study. Although the e-mail was sent to all the internal medicine program directors, it is very concerning that only 339 responded to it. On comparing with the original number, only a very small number obliged to actively participate in our survey. This indicates the disinterest and self-negligence of their own health. The other limitation of this study is its generalizability.

## Conclusions

Our study contributes to understanding of the health care needs of residents and is one of the few studies to emphasize on the primary care physician for the residents with a strong fire wall of confidentiality issues.

The programs and the program directors should consider essential steps to help residents providing them with complete insight into their personal health care. During transitional period, orientation training is pretty much mandatory in all institutions to educate them thoroughly about rules and regulations pertaining to their health needs and other significant health issues to expect a 100% outcome from them regarding their patient care. As the number of international medical graduates is almost in equal distribution compared to American medical graduates, the program directors should make tremendous effect on encroaching the cultural changes essential to help them feel comfortable during their residency period (4). With the percept of enduring the residents’ own health care practices, programs should come up with well-directed clinicians, mentors, and organizations that provide assessment of their health care, encourage clinical visits, follow-ups, and provide comprehensive services working with a strong background of confidentiality, which is one of the prominent barriers in accessing the health care.

Because residency is a unique period of intrusive stress both physically and emotionally, the additional effects involved in medical mishap may eventually decline their work performance. Residents should be encouraged to talk about their mistakes with the intention of reducing them in the future. Hence, it will be admirative if programs are obliged to provide preventive and active interventions that deal with the stress management and ultimately provide maximum patient care for the better society. Rosenthal's study that was done to know about the attitude of doctors on their work showed that physicians play a pivotal role in undertaking the responsibilities of their patients, which will be accomplished if they are at their perfect health and be fit combatively (15).

Depression during the residency period is rampant in all the programs in the United States. Not many studies have been mentioned on psychiatric problems of a resident. Therefore, significant underdiagnosed psychiatry conditions impairing the work flow need more emphasis, and more studies should come up in the future. The programs should be obliged to have a house staff preceptor to favor the residents deal with their psychiatric issues and counsel them to cope up with their routine activities.

Appropriate steps should be taken up to recognize and implement the essential health needs for the residents. Productive work from the residents can be expected by creating a friendly work environment. Discouraging the residents and physicians from working during the periods of sickness and encouraging appropriate self-care is one of the essential steps for the fruitful prospects of a physician's life. Of last and important note is that it would be fantastic if the programs enable these changes because the resident physicians always cultivate the habit to educate people what they practice.
